# Evaluation of yellow pea fibre supplementation on weight loss and the gut microbiota: a randomized controlled trial

**DOI:** 10.1186/1471-230X-14-69

**Published:** 2014-04-08

**Authors:** Jennifer E Lambert, Jill A Parnell, Jay Han, Troy Sturzenegger, Heather A Paul, Hans J Vogel, Raylene A Reimer

**Affiliations:** 1 Faculty of Kinesiology, University of Calgary, 2500 University Dr. NW, Calgary, AB T2N 1N4, Canada; 2 Physical Education and Recreation Studies, Mount Royal University, 4825 Mount Royal Gate SW, Calgary, AB T3E 6K6, Canada; 3 Food Processing Development Centre, Alberta Agriculture and Rural Development, 6309 – 45 Street, Leduc, AB T9E 7C5, Canada; 4 Department of Biochemistry and Molecular Biology, University of Calgary, 3330 Hospital Drive NW, Calgary, AB T2N 4 N1, Canada; 5 Bio-NMR Centre, Department of Biological Sciences, University of Calgary, 2500 University Dr. NW, Calgary, AB T2N 1N4, Canada

**Keywords:** Yellow pea fibre, Gut microbiota, Metabolomics, Obesity

## Abstract

**Background:**

Fibre intake among North Americans is currently less than half the recommended amount. Consumers are interested in food products that could promote weight loss and improve health. Consequently, evaluation of unique fibre sources with potential gut-mediated benefits for metabolic health warrants investigation. Our objective is to assess the effects of yellow pea fibre supplementation on weight loss and gut microbiota in an overweight and obese adult population.

**Methods/Design:**

In a double blind, placebo controlled, parallel group study, overweight and obese (BMI = 25-38) adults will be randomized to either a 15 g/d yellow pea fibre supplemented group or isocaloric placebo group for 12 weeks (n = 30/group). The primary outcome measure is a change in body fat from baseline to 12 weeks. Secondary outcomes include glucose tolerance, appetite regulation, serum lipids and inflammatory markers. Anthropometric data (height, weight, BMI, and waist circumference) and food intake (by 3-day weighed food records) will be measured at baseline and every 4 weeks thereafter. Subjective ratings of appetite will be recorded by participants at home on a weekly basis using validated visual analogue scales. At week 0 and at the end of the study (week 12), an ad libitum lunch buffet protocol for objective food intake measures and dual-energy X-ray absorptiometry (DXA) scan for body composition will be completed. Participants will be instructed not to change their exercise habits during the 12 week study. Glucose and insulin will be measured during an oral glucose tolerance test at weeks 0 and 12. Levels of lipids and CRP will be measured and inflammatory markers (adiponectin, leptin, TNF-α, IL-6 and IL-8) in the serum will be quantified using Milliplex kits. Mechanisms related to changes in gut microbiota, serum and fecal water metabolomics will be assessed.

**Discussion:**

Globally the development of functional foods and functional food ingredients are critically needed to curb the rise in metabolic disease. This project will assess the potential of yellow pea fibre to improve weight control via gut-mediated changes in metabolic health in overweight and obese adults.

**Trial registration:**

ClinicalTrials.gov (NCT01719900) Registered October 23, 2012.

## Background

The impact of obesity is increasingly felt on a global scale and includes the precipitous rise in co-morbidities such as type 2 diabetes and hyperlipidemia. In 2008 the cost associated with obesity and relevant chronic diseases was estimated to be between $4.6 and $7.1 billion in Canada, depending on the number of chronic diseases included in the estimation [1]. Of these costs, a large proportion is due to prescription drugs for management of obesity-related risk factors. For example, in 2004, pharmaceutical costs in Canada for lipid-lowering drugs alone (i.e. one singular risk factor for cardiovascular disease) was $1.39 billion [2]. In the United States, cardiometabolic risk factor clusters (including BMI > 25 and two of diabetes, hyperlipidemia or hypertension) were responsible for national medical expenditures of $80 billion, of which $27 billion went to prescription drugs [3]. Therefore, there is a distinct need for solutions for the prevention and management of obesity and its related conditions. As opposed to pharmaceuticals, targeted lifestyle interventions, particularly diet, are the safest and most economic approaches for the prevention and treatment of obesity-related conditions on a population level, and have the capacity to achieve multi-faceted health benefits.

Obesity is intimately linked to increased food intake with both quantitative and qualitative implications. In particular, fibre is a critical aspect of the diet because of its beneficial effects on numerous risk factors, including reducing plasma lipid levels, improving glucose metabolism, and enhancing satiety which helps to reduce food intake [4]. Current Institute of Medicine recommendations for dietary fibre intake are 38 g/d for adult males and 25 g/d for adult females [5]. Unfortunately, data from national nutrition surveys indicate that dietary fibre intake is currently significantly below the recommended intake level [6]. Therefore, interventions directed at increasing fibre consumption from a variety of sources are needed. Dietary fibre may also produce unique changes in gut microbiota, which independently may improve glucose tolerance, satiety, and lipid metabolism [7]. Different types of fibre and/or fibre sources may affect metabolic health in distinct ways, and therefore the diversity of fibre sources in the food supply is important [8].

### Effect of fibre on weight control and glucose tolerance

A great deal of focus has been directed towards elucidating and evaluating the beneficial metabolic effects of fibre intake on gut as well as whole-body health in humans [9,10]. The primary proposed mechanisms for how dietary fibre (specifically soluble and fermentable fibre) contributes to improved weight control and insulin sensitivity include delayed nutrient absorption, stimulation of gut hormones that regulate food intake and modulation of gut bacteria.

A recent meta-analysis of randomized controlled trials showed that increased fibre intake, whether from diets containing foods rich in fibre or soluble fibre supplements, was associated with reduced HbA1c and fasting plasma glucose in patients with type 2 diabetes [11]. The improvement in glycemic responses following a high-fibre meal has also been shown to carry through after a subsequent meal, the so-called “second meal effect” [12]. Part of the beneficial effect of dietary fibre on postprandial glucose control may be related to the release of gut peptides that influence insulin responses and food intake, such as glucagon like peptide 1 (GLP-1), peptide YY (PYY) and ghrelin. For example, feeding the prebiotic fibre oligofructose for 12 weeks reduced levels of the orexigenic hormone ghrelin and increased levels of the anorexigenic hormone PYY in overweight adults which corresponded with lower self-reported energy intake [13]. In the long-term, these changes in postprandial peptide secretion may promote weight loss through reductions in *ad libitum* food intake.

### Role of gut microbiota in obesity and insulin resistance

Members of the Firmicutes and Bacteroidetes phyla represent the majority of species in the human gut, while Proteobacteria, Actinobacteria, Chlamydiae, Spirochetes, and Fusobacteria are present in markedly lower abundance [14]. The gut microbiota phenotype can be both genetically and environmentally determined. There is some degree of genetic influence, because relatives show greater similarities in phylotypes than unrelated individuals even when these family members live in different environments [15]. However, investigations have largely been targeted towards determining how microbiota can be environmentally influenced, specifically by disease state and lifestyle interventions. For example, obese individuals have been observed to have greater proportions of Firmicutes and lesser Bacteroidetes [15,16]. A lower proportion of Bacteroidetes has also been observed as glucose tolerance worsens [17] and during progression of non-alcoholic steatohepatitis [18].

Both bacterial abundance and composition can be altered with lifestyle changes. Indeed, part of the role of intestinal bacteria in weight control has been elucidated through study of patients receiving bariatric surgery. Kong et al. [19] profiled the gut microbiota of 30 morbidly obese women before and after receiving Roux-en-Y gastric bypass surgery [19]. After the surgery, Bacteroidetes was increased and Firmicutes and bifidobacteria populations were reduced. Further, the “richness” (referring to the number of species present) was greater after surgery, and the gut microbiota composition correlated more strongly with white-adipose tissue gene expression compared to pre-surgery [19]. Weight loss produced via dietary restriction results in an increase in the relative proportion of Bacteroidetes and a reduction in Firmicutes, such that the microbiota profile becomes more similar to lean healthy control subjects [16]. Feeding specific dietary fibres can also beneficially modulate gut bacteria populations. For example, in a double-blind placebo-controlled trial in obese women, feeding 16 g/d inulin and oligofructose (50/50) vs. placebo (maltodextrin) for 3 months decreased Bacteroides, which was associated with a slight reduction in fat mass [20]. In this study, ^1^H-NMR was also performed, but there was no clear clustering effect of treatment that could be detected for gut microbial analysis or plasma/urine metabolomic profiles. Indeed, the specific metabolic function of different bacterial species is still being elucidated. However, metagenomic mapping of gut bacteria indicates a highly enriched and functionally diverse genome related to numerous pathways including metabolism of carbohydrates, energy, amino acids, and vitamins [21].

Part of the beneficial metabolic impact of gut bacteria has been attributed to the byproducts produced during fermentation of nondigestible carbohydrates (i.e. dietary fibre) by these organisms. Short-chain fatty acids (SCFA) are the primary end-products of carbohydrate fermentation by gut bacteria, and include acetate, propionate, and butyrate [22,23]. SCFA are a major energy source for colonocytes [22]. Recently, it has been discovered that SCFA are natural ligands for the G-protein coupled receptor GPR43, which is expressed in numerous tissues including immune cells, adipocytes, and endocrine cells [23,24]. While the metabolic implications of SCFA are still being elucidated, emerging data suggests that the binding of SCFA to GPR43 may improve glucose tolerance and fat metabolism. For example, in primary culture the binding of SCFA to intestinal GPR43 stimulates GLP-1 secretion by colonic L cells [25]. In animals, mice overexpressing GPR43 in adipose tissue remain lean on a high-fat diet and exhibit lower body weight, white adipose weight, and smaller adipocyte size [26]. In contrast to wild-type mice, these animals did not develop hepatic steatosis in response to a high-fat diet and had normal glucose tolerance. Finally, the GPR43 mice also displayed greater energy expenditure and increased fat oxidation [26]. While these findings are preliminary, the implication is that SCFA may have important effects on the host organism beyond energy production in the gut.

### Pea fibre as a novel and sustainable fibre source

Recently, the interest in pulse crops (e.g. dried beans and peas) as sources of dietary fibre has increased [27]. Yellow peas are a native Albertan pulse crop [28] with the potential to have beneficial effects on plasma lipids, glucose metabolism, and weight control. Pea hulls are comprised of ~82% fibre making them an excellent source of dietary fibre that could be incorporated into food products [29].

To date, few studies have examined the metabolic effects of pea fibre in animal models. Briefly, in rats with diet-induced glucose intolerance, feeding dry pea seed coats improved glucose tolerance as well as fasting and glucose-stimulated insulin levels [30]. Investigation of skeletal muscle expression of proteins involved in insulin signaling suggested that pea seed coat consumption increased glucose transport. Finally, pea seed coat consumption also appeared to reduce markers of oxidative stress [30]. In hypercholesterolemic Golden Syrian hamsters, feeding whole pea flour or fractionated pea flour (hulls only) did not reduce plasma lipid levels, but decreased fasting glucose and insulin concentrations, and showed a statistical trend towards reducing body fat [31]. More recently, we have demonstrated that yellow pea fibre reduces glycemia in diet-induced obese rats while yellow pea flour (rich in pea protein) reduces adiposity (Eslinger et al., Submitted). Both pea fibre and pea flour reduced the percent of fecal Firmicutes.

The state of the literature regarding metabolic effects of pea hull fibre specifically in humans is limited to few studies. In an acute meal setting, adding pea fibre (10 g) to a low-fibre (2.8 g) test meal resulted in lower postprandial plasma cholesterol levels compared to soybean fibre in normolipidemic men, but postprandial glucose, insulin, and plasma triglyceride were not affected by fibre type [32]. Similarly, isoenergetic high-fibre (26 g) and low-fibre (9 g) meals produced similar postprandial glucose and insulin in healthy normal men, however plasma free fatty acids were better suppressed after the high-fibre meal which contained pea fibre baked into wheat bread [33]. Conversely, in one study of healthy adults, pea fibre (15 g) incorporated into a mixed test meal significantly reduced the postprandial glucose incremental area under the curve as compared to sugar beet fibre and wheat bran, while addition of all fibres reduced the postprandial insulin response [34].

As opposed to the effects of acute ingestion of pea fibre, the metabolic effects of consuming diets containing foods fortified with pea fibre are also limited to a few studies. In one study, addition of pea hull fibre to existing foods (resulting in an average of 3 g/d pea fibre) increased stool frequency in elderly residents of a long-term care facility, representing a safe, economical, and non-invasive alternative to relieve constipation in the elderly [35]. Feeding young healthy adults a low-fibre diet or a low-fibre diet supplemented with 33 g/d pea fibre product (corresponding to 20 g dietary fibre) in a randomized cross-over study showed no differences in fasting cholesterol levels however triglyceride levels in the plasma and those arising from the liver were reduced in the pea fibre supplemented group [36]. In addition, incorporation of the fibre into breakfast and lunch meals resulted in significantly lower plasma and chylomicron triglyceride responses, as well as plasma insulin concentrations. Incorporation of whole yellow pea flour into baked products reduced postprandial glycemia in healthy men and women compared to products containing whole wheat flour [37]. Finally, in a population who could potentially benefit the most from dietary fibre provision, providing baked products containing pea fibre (12 g/d of fibre) to overweight hypercholesterolemic adults for 28d reduced fasting insulin concentrations and improved postprandial glucose responses after a standardized breakfast meal [38]. Given the limited state of evidence for the potential benefits of pea fibre in individuals at-risk for obesity and metabolic syndrome, clinical trials specifically examining the clinical outcomes and mechanisms related to pea fibre consumption are warranted.

### Specific objectives

This project proposes to perform a comprehensive investigation of the effects of pea fibre supplementation on 1) weight loss, 2) glucose control and features of the metabolic syndrome, 3) food intake and satiety, and 4) gut microbiota and serum and fecal water metabolites in free-living overweight and obese adults. The combination of anthropometric measurements with plasma metabolomics and analysis of fecal microbiota will provide unique information on the whole-body and *in vivo* effects of pea fibre consumption in overweight adults.

Given the research aims, the primary outcome is change in body fat mass from baseline to 12 weeks. Our secondary outcomes include glucose tolerance, serum lipids and inflammatory markers, and objective and subjective measures of food intake and appetite. To better understand the mechanisms by which yellow pea fibre may impact metabolism, we will also undertake exploratory mechanistic studies on changes in intestinal microbiota and *in vivo* metabolism assessed using metabolomics analysis.

It is first hypothesized that the group consuming the pea fibre will have a greater reduction in body fat compared to the control group. Second, it is predicted that at follow-up the pea fibre group will have lower fasting insulin concentrations and reduced glucose and insulin excursions during an oral glucose tolerance test (OGTT). Third, it is expected that the pea fibre group will have greater ratings of fullness and a reduction in *ad libitum* energy intake compared to control. Finally, it is anticipated that supplementation of pea fibre will beneficially alter gut microbiota and induce a unique serum and fecal water metabolite signature reflective of the changes in body fat mass.

## Methods/design

### Design, participants, and setting

This study will utilize a double-blind, placebo-controlled, parallel group design in which 60 overweight and obese adults (age 18-70 y; BMI 25-38 kg/m^2^) will be randomized to either a placebo (n = 30) or pea fibre supplemented (n = 30; 15 g/d yellow pea fibre) group for 12 weeks. Randomization will be carried out using computer generated numbers and stratified according to age, sex, and BMI.

Adults with a stable body weight for at least 3 months prior to the study, defined as weight loss or gain of <3 kg within preceding 3 months to enrollment, will be eligible to participate. Exclusion criteria include: concomitant use of any weight loss medication (e.g. Orlistat) or diet/exercise regime designed for weight loss; use of corticosteroids, anti-depressants, or anti-epileptic, lipid-lowering, and diabetes medications; previous bariatric or other intestinal surgery; pregnancy or lactation; use of bulk laxatives or probiotic/prebiotic supplements; chronic use of antacids; use of antibiotics within the preceding 3 months of enrollment; clinically significant cardiovascular or respiratory disease; liver disease; alcohol or drug dependence; body weight >350 lb; active malignancy; and chronic infections. Eligibility will be assessed by the researchers using a screening questionnaire and phone interview.

**Figure 1:**
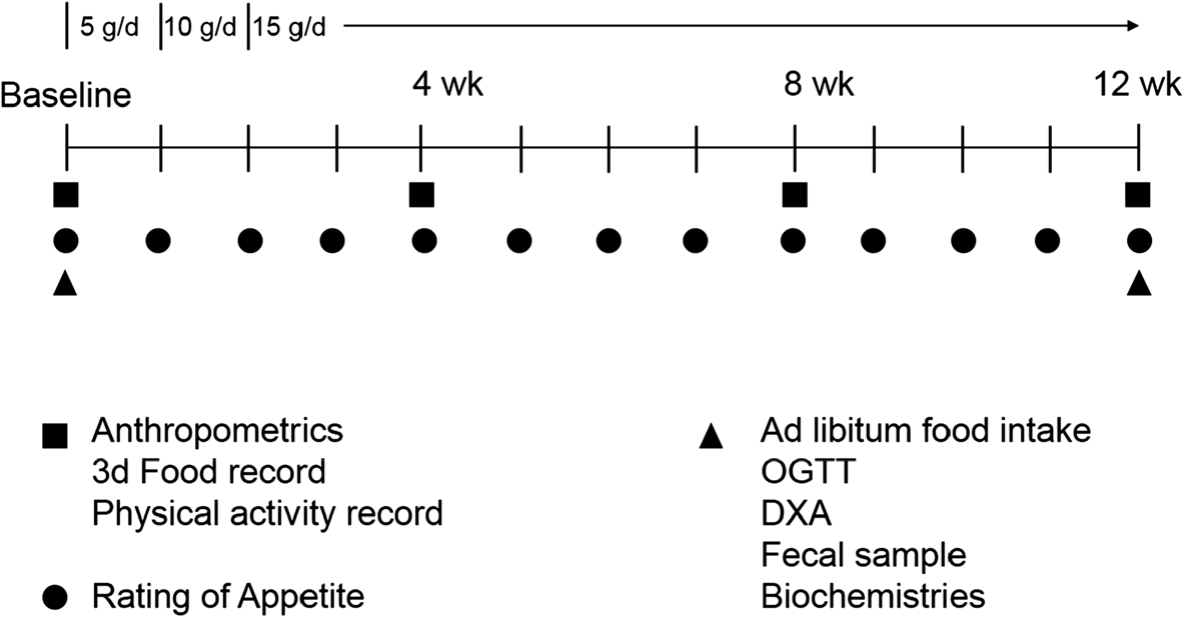
Overall study design of the fibre supplementation intervention.

The overall study design is described in Figure 1, and the outcome measures are described individually below. Anthropometric data (weight, BMI, waist circumference) and food intake (3-day weighed food record) will be collected at baseline and at 4 week intervals. Subjective ratings of appetite will be measured using validated visual analogue scales (VAS) by the subjects at home on a weekly basis. At baseline and follow-up (12 weeks), body composition will be measured by DXA, and an oral glucose tolerance test will be performed to assess glucose tolerance. Plasma inflammatory markers (including TNF-α and CRP), lipids (total, LDL- and HDL-cholesterol, and triglyceride), and satiety hormones (including GLP-1, PYY, ghrelin and leptin) will be measured using methods already established in the laboratory and described below. Subjects will be provided with an *ad libitum* lunch buffet to measure objective food intake. Serum samples will undergo metabolomics analysis using proton nuclear magnetic resonance spectroscopy (^1^H-NMR) [39]. ^1^H-NMR will allow us to measure global metabolic responses which will complement the plasma biomarkers concurrently measured. Stool samples will be collected for analysis of gut microbiota and for fecal water metabolomics using ^1^H-NMR.

### Dietary intervention and novel food product development

Participants in the active arm of the intervention will consume biscuits containing 5 g of pea fibre per serving three times per day, within 30 min of their 3 largest daily meals. The control group will consume an isocaloric amount of a control biscuit that is similar in taste and texture but contains no pea fibre. To minimize gastrointestinal discomforts associated with a rapid increase in fibre intake, the dose will be slowly increased during the first 3 weeks of the study (week 1 = 5 g/d; week 2 = 10 g/d; week 3 = 15 g/d). The biscuits will be formulated with a low energy content and will be designed and produced at the Food Processing Development Center of Alberta Agriculture and Rural Development (Leduc, AB). Biscuits will be produced in Leduc and shipped to Calgary. Each subject will record the time the product was consumed on a calendar provided by the investigators. Subjects will be instructed to return all biscuit packages and any unconsumed biscuits for counting to assess compliance and actual intake. Subjects will maintain their *ad libitum* habitual food intake during the intervention, which will be monitored via completion of monthly 3-day weighed food records. The study is designed to examine the effects of pea hull fibre supplementation in a real-life setting, independent of any other diet or exercise intervention; therefore, subjects will be encouraged to maintain their regular lifestyle, to eat until comfortably full and not to consciously try to gain or lose weight throughout the study. At the end of the study an informal interview will be conducted to assess whether or not the subjects remained unaware of the treatment they received.

### Laboratory analyses and outcome measurements

#### ***Anthropometrics and dual X-ray absorptiometry***

Height, weight, BMI, and waist circumference will be measured at baseline and every 4 weeks thereafter during clinical visits with research staff. Body weight will be measured using a standard balance beam scale. Waist circumference will be measured at the level of the umbilicus using a tape measure by the same research staff member at all visits. Percent fat mass and lean mass will be measured using a whole body dual-energy x-ray absorptiometry (DXA) scan (Hologic QDR 4500, Hologic, Inc., Bedford, MA, USA). These results will be used in conjunction with Hologic QDR software to determine lean mass, bone mineral content and fat mass. Bone mineral density (BMD) and percent fat will also be determined.

#### ***Food record***

Food and beverage intake will be assessed using 3-day weighed food records at baseline and every four weeks thereafter using food scales and standardized forms provided to the subjects. As described above, subjects will not be prescribed specific diets and will instead be encouraged to maintain their habitual intake. Prior to the start of the study all subjects will attend a training session delivered by a Registered Dietitian in which they will be instructed on the use of the food scale and how to record their food intake. Subjects will then be instructed to weigh and record all food consumed for 2 weekdays and 1 weekend day. This information will be analyzed with FoodWorks software (The Nutrition Company, Long Valley, NJ). Participants will be instructed to maintain their current level of physical activity throughout the study. Physical activity will be monitored using the Godin’s Leisure Time Exercise Questionnaire which will be completed at baseline, 4, 8 and 12 weeks.

#### ***Subjective and objective assessments of appetite***

Subjective ratings of appetite will be recorded by participants at home on a weekly basis using a validated 100 mm VAS [40,41]. The questions are:

1) “How hungry do you feel?” anchored by “I am not hungry at all” and “I have never been more hungry”.

2) “How satisfied do you feel?” anchored by “I am completely empty” and “I cannot eat another bite”.

3) “How strong is your desire to eat” anchored by “I have no desire to eat” and “I have a great desire to eat”.

4) “How full do you feel?” anchored by “Not at all full” and “Totally full”.

5) “How much do you think you could eat?” anchored by “Nothing at all” and “A lot”.

At baseline and end of the study (week 12), an ad libitum lunch buffet protocol will be performed to objectively assess food intake. This buffet-style provision of foods has been shown to be highly reproducible for the measurement of energy intake and macronutrient preferences [42-44]. The ad libitum lunch will be served following the OGTT (3 hours after taking the oral glucose drink). The subjects will be instructed to eat until "comfortable satisfaction," and each subject's energy intake will be measured. Conversation between subjects will be monitored such that normal conversation will be allowed as long as the topics do not involve food, appetite, or related issues [43]. The lunch will consist of two food choices. The first will be a savory food (i.e. cheese pizza) and the second a sweet food (i.e. cookies) [45,46]. The energy content of the food choices will be pre-determined, and subjects will be given a pre-weighed amount of the two choices and allowed to eat until satisfied. Though the food quantity will be pre-determined, each item will be provided in an amount corresponding to a reasonable surplus, so no researcher-imposed limit is placed on the subject’s consumption. Upon meal completion, the remaining food not consumed by the subjects will be weighed by research staff. Subjective ratings of appetite using the VAS will be collected immediately before and after the ad libitum meal. The acceptability of the meal will be assessed with the following question, “How acceptable or pleasing did you find the meal?” anchored by “Not at all acceptable” and “Highly acceptable”.

#### ***Oral glucose tolerance test***

At baseline (day 0) and follow-up (wk 12) after an overnight fast, glucose tolerance will be assessed with an OGTT. A cannula will be inserted into the antecubital vein to allow for repeated blood draws. Following an overnight fast, a baseline blood sample will be drawn. Subjects will be given a 75 g oral glucose drink and additional blood samples taken at time = 30, 60, 120 and 180 minutes by registered nurses. Blood will be centrifuged and plasma frozen at -80°C for subsequent analyses. Glucose will be quantified using a glucose trinder assay (Stan Bio, Boerne, TX, USA).

#### ***Plasma lipids and inflammatory markers***

At baseline and follow-up a fasting blood sample will be taken for measurement of plasma HbA1c, lipids and CRP. Samples will be sent to Calgary Laboratory Services for analysis. Serum inflammatory markers (adiponectin, leptin, TNF-α, IL-6 and IL-8) will be quantified using Milliplex kits (Millipore, Billerica, MA, USA).

#### ***Satiety hormones***

At each of the five time points during the OGTT, additional blood samples will be collected for measurement of satiety hormones. Blood will be drawn into a cooled EDTA vacutainer tube containing diprotinin-A (0.034 mg/ml blood; MP Biomedicals, Irvine, CA); Sigma protease inhibitor (1 mg/ml blood; Sigma Aldrich, Oakville, ON, Canada) and Roche Pefabloc (1 mg/ml of blood; Roche, Mississauga, ON, Canada) [13]. Blood will be centrifuged within 30 min and plasma analyzed for ghrelin (active), insulin, leptin, glucose-dependent insulinotropic polypeptide (GIP) (total), GLP-1 (active), and PYY (total) concentrations using a Human Gut Hormone Panel Milliplex Kit (Millipore, St Charles, MO).

#### ***Gut microbiota***

Stool will be collected at baseline and follow-up for analysis of gut microbiota. Subjects will be instructed on proper methods for stool sample collection and all materials will be provided in a convenient specimen collection kit. Each subject will collect 2 tablespoons of stool into a pre-labeled sterile container. The container will be sealed, placed in a biohazard bag, and immediately stored in a standard home freezer (-20C). The specimen will be transported to our research lab within 4 days of collection in a styrofoam container on an ice pack.

For analysis of gut bacteria species, total bacterial DNA will be extracted from the frozen fecal samples using the QIAamp DNA Stool Mini Kit (Qiagen, Mississauga, ON). Bacterial DNA concentrations will be measured using the Pico-Green DNA Quantification Kit (Invitrogen, Carlsbad, CA). Microbiota will be quantified using quantitative PCR according to our previous work [47] using the BioRad iCycler (BioRad Inc., Mississauga, ON) and group-specific primers. Briefly, amplification and detection will be conducted in 96-well plates with SYBR Green 2 × qPCR Master Mix (BioRad). Group specific primers have been previously published [47]. The 16S rRNA gene copies value will be calculated according the following webpage: http://cels.uri.edu/gsc/cndna.html using average genome sizes. Standard curves will be normalized to the copy number of the 16S rRNA gene obtained from the following webpage: http://rrndb.umms.med.umich.edu/.

#### ***Serum metabolomics***

Metabolomics analysis provides a snapshot of an organism’s current metabolic profile. Beyond measurement of single metabolites, applying principal component analysis (PCA) to metabolomics data allows for the relationships between variables to be determined, thereby identifying a metabolic signature [48]. Sample preparation and metabolomics analysis will be performed according to our previous work [39]. Briefly, following sample preparation, including filtering and pH standardization, all NMR experiments will be performed on a Bruker Advance 600 spectrometer (Bruker Biospin, Milton, Canada). Processed spectra will be imported into Chenomx NMR Suite software (Edmonton, AB) for metabolite identification and quantification. In order to describe the metabolite changes in the context of other physiological variables, metabolomic data will be integrated with the other biological variables assessed (blood biochemistry, satiety hormones, body composition) using O2PLS-DA (orthogonal partial least squares discriminatory analysis) modeling [39,49].

#### ***Fecal water metabolomics***

The fecal samples collected at baseline and 12 weeks will be used for fecal water ^1^H-NMR metabolomic analysis. The NMR sample preparation protocol will be based on previously published work that has been shown to maximize retrieval of SCFAs from the fecal samples [50]. ^1^H-NMR spectra will be acquired on a Bruker Advance 600 spectrometer (Bruker Biospin, Milton, Canada) operating at 600.22 MHz, and multivariate analysis of metabolic profiles will be performed using SIMCA-P + 12.0.1 software (Umetrics). The PCA and O-PLS-DA models of the fecal water extracts are expected to show separation between the control and pea fibre groups based on metabolite concentrations involved in gut microbiota activity resulting from fibre fermentation.

### Sample size calculation

Sample size was calculated based on anticipated changes in body fat using data from our previous study of prebiotic fibre supplementation in overweight and obese adults [13]. The expected change in body fat in the pulse fibre group is -1.11 kg whereas the expected weight change in the control group is +0.20 kg, with a standard deviation of ±1.6 kg. Assuming an alpha = 0.05 and power = 0.80 (80%) we would need to recruit 24 subjects in each study arm. An additional 6 patients per group will be added to account for the expected 20% loss to follow up for a total of 30 patients per group.

### Statistical analysis

Data will be analyzed using SPSS statistical program (IBM Software, Armonk, NY) with results considered statistically significant (2-sided) at P ≤ 0.05. Values with skewed distribution will be logarithmically transformed prior to analysis. Baseline characteristics will be compared between groups using chi-square for categorical variables and *t* tests for continuous variables. Primary analysis will be performed on an intent-to-treat basis, regardless of subject compliance. The primary outcome measure is change from baseline to 12 weeks in body fat. ANCOVA, with testing for confounding factors (sex) and potential covariates (BMI, age) will be used to assess the difference between groups at weeks 0 and 12. Should the main effect of diet be significant, Tukey’s multiple comparison test will be used. Secondary outcomes measured at 0 to 12 weeks (e.g. satiety hormones, glycemia, insulinemia, food intake) will be analyzed with ANCOVA as above. For variables measured over time (e.g. body weight, satiety hormones and glucose at 0, 30, 60, 120 and 180 minutes), a mixed model of repeated measures ANCOVA will be applied to examine the effect of treatment, time and their interaction. Our planned secondary analysis will be per protocol with all subjects who complete the 12 week intervention and are compliant based on consumption of ≥80% the product doses. Cases with missing outcome data will be excluded from analysis.

For metabolomics analysis, in addition to univariate tests, multivariate analysis will conducted using SIMCA-P + 12.0.1 software (Umetrics, Sweden). Data will be reprocessed by mean-centering and unit variance scaling. A supervised partial least squares discriminant analysis (PLS-DA) approach will be used to compare the variance of metabolite concentrations between the two sample classes (diet: control and pea fibre). A supervised orthogonal partial least squares analysis will be used (control versus pea fibre) for a direct comparison of the variance between diet type (Y variable) and metabolite concentrations (X variable). The results from the metabolomics analysis will also be combined with the plasma satiety hormones and other biological measurements and regressed to the diet type using O2-PLS-DA (orthogonal PLS-DA).

### Ethics

This proposal has been approved by the Conjoint Health Research Ethics Board of the University of Calgary. Voluntary, written informed consent will be obtained from each participant.

## Discussion

The proposed study will determine whether increased consumption of fibre derived from yellow peas enhances weight loss and specifically body fat loss compared to control. In addition, the effect of pea fibre on glucose tolerance, appetite, serum lipids and inflammatory markers will also be determined. Mechanisms related to changes in gut microbiota and serum and fecal water metabolites will be examined. The goal of this research is to provide evidence for the potential role of pulse-derived fibre in weight management. This work is important in light of the growing interest by consumers, food manufacturers and health care professionals in functional foods that impact long-term health.

The global functional food market has expanded substantially in recent years, and is expected to continue growing in the coming years [51,52]. For example, from 2000 to 2006 the global functional food market increased by 68% [51]. This growing market for functional foods has spurred development of research-oriented businesses that in 2004 generated annual revenues of $2.9 billion and exported $545 million worth of products in Canada alone [53]. As compared to drug development, functional food development has the advantages of shorter development periods and lower development costs [54]. Development of the functional food product sector is beneficial for consumers, the agri-food sector, and public health [51,54]. Indeed, a significant proportion of research and development in major food companies is directed towards developing “healthful” foods [55]. In particular, food products aimed at gut health dominate the functional food markets in Japan and Europe, including both probiotic- and prebiotic-fortified foods [54].

In the current study, pulse fibre will be incorporated into a cookie-type biscuit that will be consumed 30 minutes prior to a meal, thereby acting as a meal pre-load. Consuming the biscuit between meals has implications for snacking behaviors and potential new snack foods. Modifying snack options presents a key area to expand research in dietary supplements as snacking is prevalent [56]. Not only does snacking displace healthy nutrient-dense foods but the energy density of snacks has also increased over time such that the contribution of snacking to overall energy intake is substantial (24% by one estimation) [56,57]. However, snacking is not necessarily a negative eating behavior, because it depends on food choices. For example, one study found that high-frequency snack consumption occurred in individuals with both healthy and unhealthy dietary patterns [58]. Given the increased prevalence of snacking in combination with the increase in food consumption away from home and desire for convenient food products [59,60], snack foods represent an ideal behavior to modify by replacing currently consumed food options with more nutrient-dense options that are attractive and palatable, and that also have the potential to positively affect health outcomes. Ideal snacks would be those which are healthy and balanced in macronutrient proportions; provide functional nutrients that individuals may not otherwise consume; and are portable without requirement for preparatory methods, so they can be incorporated easily into daily consumption at home or work.

Ultimately, this project will determine whether yellow pea fibre has the potential to be incorporated as a functional food ingredient in products aimed at weight management and metabolic health.

## Abbreviations

BMI: Body mass index; CRP: C-reactive protein; DXA: Dual energy x-ray absorptiometry; GL: Glycemic load; GLP-1: Glucagon-like peptide-1; 1H-NMR: Proton nuclear magnetic resonance; HbA1c: Hemoglobin A1C; OGTT: Oral glucose tolerance test; O2PLS-DA: Orthogonal partial least squares discriminatory analysis; PYY: Peptide YY; TNF-α: Tumor necrosis factor alpha; VAS: Visual analogue scale.

## Competing interests

The authors declare that they have no competing interests. This work is partially funded by the Alberta Pulse Growers Commission.

## Authors’ contributions

JEL drafted the manuscript. JAP participated in design of the study related to gut microbiota analysis and dietary fibre dosing, and provided manuscript revisions. JH and TS participated in design of the study related to food product development and packaging, and provided manuscript revisions. HAP participated in design of the study related to gut microbiota and metabolomics analysis and provided manuscript revisions. HJV participated in design of the study related to metabolomics analysis and provided manuscript revisions. RAR conceived of the study and was involved in the overall design, and helped to draft the manuscript. All authors read and approved the final manuscript.

## Authors’ information

HJV holds the Armstrong Chair for Molecular Cancer Research.

## Pre-publication history

The pre-publication history for this paper can be accessed here:

http://www.biomedcentral.com/1471-230X/14/69/prepub
